# Multifaceted functions and roles of HBZ in HTLV-1 pathogenesis

**DOI:** 10.1186/s12977-016-0249-x

**Published:** 2016-03-15

**Authors:** Guangyong Ma, Jun-ichirou Yasunaga, Masao Matsuoka

**Affiliations:** Laboratory of Virus Control, Institute for Virus Research, Kyoto University, Kyoto, Japan

**Keywords:** HTLV-1, HBZ, Tax, Viral oncogenesis, Regulatory T cell

## Abstract

Human T cell leukemia virus type 1 (HTLV-1) is an oncogenic retrovirus responsible for the development of adult T-cell leukemia (ATL). Although HTLV-1 harbors an oncogene, *tax*, that transforms T cells in vitro and induces leukemia in transgenic mice, tax expression is frequently disrupted in ATL, making the oncogenesis of ATL a bit mysterious. The HTLV-1 bZIP factor (HBZ) gene was discovered in 2002 and has been found to promote T-cell proliferation and cause lymphoma in transgenic mice. Thus HBZ has become a novel hotspot of HTLV-1 research. This review summarizes the current findings on HBZ with a special focus on its potential links to the oncogenesis of ATL. We propose viewing HBZ as a critical contributing factor in ATL development.

## Background

Human T-cell leukemia virus type 1 (HTLV-1) is the first human retrovirus to have been identified (in the early 1980s), and it was later demonstrated to be the causative agent of adult T-cell leukemia (ATL), an aggressive cancer of peripheral CD4 T cells [1, 2]. HTLV-1 is able to infect various cell types in vitro, yet the HTLV-1 provirus is predominantly detected in CD4 T cells in vivo [3]. The CD4 T cell tropism of HTLV-1 is likely due to selected expansion of infected CD4 T cells rather than a receptor preference, because the HTLV-1 receptor, glucose transporter 1 (GLUT1) is ubiquitously expressed [4, 5].

**Fig. 1:**
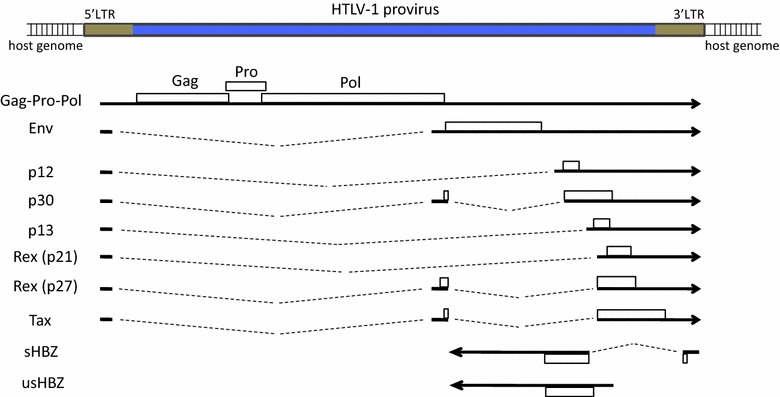
HTLV-1 provirus, mRNAs and proteins. HTLV-1 provirus is shown on *top in blue* with both LTRs painted in *brown*. Below, the transcripts and proteins encoded by a complete HTLV-1 provirus are shown. Sense transcripts are portrayed as *black arrows* with *arrowheads* pointing to the right, while antisense transcripts run in the opposite direction. Spliced exons are shown in *solid black lines* while introns are shown as *dotted lines*. Proteins derived from respective mRNAs are shown as *empty squares*

The HTLV-1 provirus is 9 kb long and has multiple coding regions flanked by two identical 750-bp long terminal repeats (LTRs) in the 5′ and 3′ ends (Fig. 1), both of which are composed of unique 3′ (U3), repeat (R) and unique 5′ (U5) regions. The 5′ LTR serves as the promoter for all structural genes and most accessary and regulatory genes, including the gene for the transactivator Tax, which upregulates 5′ LTR activity by recruiting cAMP response element-binding protein (CREB) to three viral CREB-responsive element (vCRE) tandem repeats in the 5′ LTR [6]. Transcriptional coactivators such as CBP/p300 and P/CAF are also recruited to vCRE by Tax [6]. The 3′ LTR is able to initiate transcription from the negative strand of the provirus and serves as the promoter for the only antisense transcript of the virus, HTLV-1 basic leucine zipper factor (HBZ) [7–9].

Although most HTLV-1 infected individuals remain lifelong asymptomatic carriers, approximately 5 % of them will develop ATL after a long latency of decades [10]. HTLV-1 also causes several inflammatory diseases such as uveitis, dermatitis and a neurodegenerative disorder called HTLV-1-associated myelopathy/tropical spastic paraparesis (HAM/TSP) [11].

## Review

### The *HBZ* gene

Tax is of crucial importance for its unique ability to drive HTLV-1 replication and to immortalize T cells [12] and thus has long been thought to be the main causal factor of ATL. However, it has been reported that Tax expression is frequently inactivated in ATL cases by either abortive mutations in the *tax* gene or DNA methylation of the 5′ LTR [13–16]. In addition, a defective provirus with the 5′ LTR partially or completely deleted has been found in up to 40 % of ATL cases [17, 18]. Host immunosurveillance by cytotoxic T lymphocytes (CTLs) is thought to be responsible for the loss of Tax expression, since Tax protein is a major target of CTLs [19]. In contrast to the 5′ LTR, the 3′ LTR remains intact and non-methylated—and the *HBZ* gene harbors no abortive mutations and is consistently expressed in ATL patients and HTLV-1 infected individuals [18, 20, 21]. Furthermore, *HBZ* mRNA abundance positively correlates with HTLV-1 proviral load in asymptomatic carriers (AC), HAM/TSP and ATL patients [22–24]. The distinct expression patterns of *HBZ* and *tax* suggest that they have different roles in the course of HTLV-1 pathogenesis.

The *HBZ* gene has two transcription isoforms: an unspliced (usHBZ) form and a spliced (sHBZ) form. usHBZ was discovered in 2002 [8] and early publications on HBZ were exclusively based on usHBZ. The alternative transcript, sHBZ, was first reported in 2006 [25–27]. These two transcripts have different 5′ untranslated regions (UTRs) and differ slightly in the 5′ region of their coding sequences (CDS) (Fig. 1). Consequently, the usHBZ and sHBZ proteins have almost identical sequences except for the first several amino acids (MAAS for sHBZ and MVNFVSA for usHBZ). Previous studies have shown that usHBZ and sHBZ exhibit similar functions. However, since sHBZ is more abundantly expressed in infected cells [9, 22], current studies are mostly focused on sHBZ. This review mainly addresses the functions of sHBZ.

The transcription of sHBZ initiates from the U5 and R regions of the 3′ LTR [25, 27], and the whole 3′ LTR likely serves as a TATA-less promoter of sHBZ [9]. sHBZ transcription terminates at a classical polyadenylation signal downstream [27]. Three vCRE [28] and three specificity protein 1 (Sp1) binding sites [9] have been discovered in the 3′ LTR, and they seem to be critical for its promoter activity. Due to the presence of vCRE, the activity of the 3′ LTR is enhanced by Tax via a CREB-dependent mechanism [28]. HBZ, by recruiting JunD to the Sp1 sites, also enhances the activity of the 3′ LTR [29].

It is interesting that the activity of the 3′ LTR seems to respond to some stimuli in an opposite way from that of the 5′ LTR. It has been reported that two Tax antagonistic cellular proteins, TCF1 and LEF1, significantly inhibit Tax-mediated 5′ LTR activation but slightly enhance 3′ LTR activation [30]. In addition, valproic acid (VPA), a deacetylase inhibitor, is reported to have opposite effects on the 3′ and 5′ LTRs, in that it represses HBZ expression but increases Tax expression [31].

### Functions of HBZ protein

**Fig. 2:**
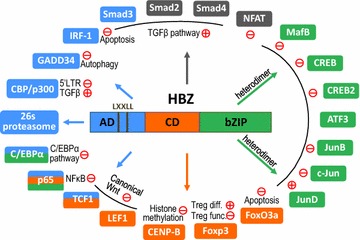
Cellular proteins that interact with HBZ. The three domains of sHBZ are portrayed as *squares in* *three different colors* while the cellular binding partners of HBZ are demonstrated as *squircles with colors* corresponding to that of AD, CD or bZIP domain of HBZ that they interact with. Cellular proteins that bind to more than one domain of HBZ are painted in *multiple colors* whereas those lacking such information are painted in *dark grey*. The major pathways that these interactions have impact on, either positive (+) or negative (−), are noted close to respective cellular proteins while more detailed description can be found in the text. Some of the interactions are not discussed in the text due to space limitations, which include 26s proteasome [93], CENP-B [94], C/EBPα [95] and IRF-1 [96]. For Foxp3, HBZ enhances transcription from the Foxp3 promoter (Treg differentiation: diff.) while it suppresses function of Foxp3 (Treg function: func.)

HBZ is a nuclear protein [32–35] and comprises an activation domain (AD) in the N-terminus, a central domain (CD), and a basic leucine zipper (bZIP) domain in the C-terminus (Fig. 2). The N-terminus of HBZ was found to possess transactivating potential when fused with the DNA-binding domain of GAL4 and therefore termed AD [8]. Within the AD of HBZ, two LXXLL-like motifs have been identified and shown to bind to the KIX domain of CBP/p300 [36], well-known transcription coactivators that are involved in a variety of cellular functions [37]. These LXXLL motifs are also required for HBZ to activate TGF-β/Smad signaling, which is critical for HBZ-induced Foxp3 expression [38]. The bZIP domain enables HBZ to hetero-dimerize with cellular bZIP proteins of the AP1 superfamily [39], such as CREB2 [8], c-Jun [40, 41], JunB [40], JunD [29, 42], CREB [43], MafB [44] and ATF3 [45] (Fig. 2). In most cases the HBZ/AP1 hetero-dimerization impairs the association of AP1 proteins with their responsive DNA elements [8, 40, 41, 43, 44] but in some cases dimerization can instead result in enhanced DNA binding—as is the case for JunD [29, 42]. It should be noted that although HBZ protein is modified by phosphorylation, acetylation or methylation, a recent report demonstrates that none of these post-translational modifications likely affect its function [46].

#### HBZ and HTLV-1 infectivity

When overexpressed, HBZ is found to repress the formation of the transactivation complex composed of Tax, CREB, CBP/p300, P/CAF and vCRE and subsequent activation of the HTLV-1 5′ LTR in vitro [6, 12] by hetero-dimerizing with CREB proteins via the bZIP domain [8, 40, 43] and interacting with CBP/p300 via the LXXLL motifs [36]. However, when HBZ expression was knocked out (KO) from an HTLV-1 infectious clone by introducing a premature stop codon (clone termed HBZ-KO), virus production was not affected [47, 48]. HBZ knockdown in an HTLV-1 infected cell line did not affect viral replication [49], suggesting a distinct effect of endogenous HBZ on viral infectivity. Interestingly, when this HBZ-KO HTLV-1 clone was used to infect rabbits, proviral copies were significantly suppressed, indicating a positive role of HBZ in HTLV-1 infectivity or proliferation of infected cells in vivo [47]. In addition, the same HBZ-KO HTLV-1 clone has also been tested in a monkey infection model [48]. All four monkeys were successfully infected, but it was very difficult to determine whether this mutant would demonstrate an impaired infectivity due to the lack of a wild-type HTLV-1 clone as control in the monkey experiments. Nevertheless, an intriguing finding, which was not observed in rabbits, is that the abortive mutations in the HBZ-KO HTLV-1 clone gradually reverted to wild type as infection prolonged, until revertant clones with intact HBZ genes became dominant. This observation might imply a fairly stringent requirement for wild type HBZ in HTLV-1 infection in monkeys, although it should be confirmed in larger number of monkeys. Therefore, despite inhibiting HTLV-1 replication in vitro, HBZ seems to be indispensible for HTLV-1 infectivity or proliferation of infected cells in vivo.

#### HBZ maintains a persistent HTLV-1 latent infection

Tax is known to activate various cellular signaling pathways including the well-known NFκB pathway [50]. In contrast, HBZ does not affect non-canonical NFκB pathway, and HBZ inhibits the canonical NFκB pathway by repressing the DNA-binding potential and inducing proteasomal degradation of p65 [51]. The significance of this function of HBZ was obscure until the recent discovery that Tax-mediated NFκB hyperactivation leads to senescence in HeLa cells—senescence which is alleviated by HBZ because of its ability to inhibit canonical NFκB activation. HBZ completely abrogated canonical NFκB activation by Tax without affecting its activation of the 5′ LTR, and thereby allowed cells to overcome Tax-triggered senescence and grow continuously [52].

The proper expression of HTLV-1 structural proteins relies on Rex-mediated nuclear export of viral mRNAs [53]. In contrast, the nuclear export of accessory and regulatory viral mRNAs, especially *HBZ*, is Rex-independent [54, 55]. Interestingly, HBZ can block the Rex-mediated nuclear export of viral structural transcripts such as Gag-Pol and thus prevent their translation [56], maintaining a latent HTLV-1 infection without the production of virus particles. Thus HBZ does not support productive HTLV-1 replication, but rather appears to maintain HTLV-1 persistence by suppressing senescence and inducing viral latency.

#### HBZ promotes proliferation of T cells

Among the AP1 superfamily proteins that can interact with HBZ, ATF3 and JunD are upregulated at the transcriptional level in ATL [45, 57]. ATF3 is a transcription factor that belongs to the ATF/CREB family. ATF3 has bimodal functions in oncogenesis, because on the one hand it activates p53 signaling and acts as a tumor suppressor, but on the other hand it is upregulated in some cancers and promotes proliferation [45]. Interestingly, HBZ impedes the p53-enhancing function of ATF3 that is deleterious to ATL development, but HBZ does not hinder the growth-promoting effect of ATF3 [45]. The expression of JunD is increased in cutaneous T-cell lymphomas as well as in ATL [58]. HBZ not only interacts with JunD and enhances its transcriptional activity, but also induces JunD expression in NIH3T3 cells. Importantly, HBZ-induced cellular proliferation can be impaired by JunD knockdown, indicating that HBZ indirectly promotes proliferation via JunD [29].

Two studies suggest that HBZ also employs certain autocrine/paracrine pathways to enhance ATL cell proliferation [59, 60]. HBZ upregulates the transcription of the noncanonical Wnt ligand Wnt5a while suppressing the canonical Wnt pathway that is detrimental to ATL cell growth. Wnt5a enhances both proliferation and migration of ATL cells [59]. Recently another study showed that HBZ upregulates brain-derived neurotropic factor (BDNF) expression via enhancing its promoter activity [60]. Upregulation of BDNF and its receptor, tropomyosin receptor kinase B (TrKB), further promotes ATL cell proliferation [60].

#### HBZ inhibits apoptosis and autophagy

HBZ hinders activation-induced cell death in T cells. The expression of Bim, a pro-apoptotic gene, is greatly suppressed by HBZ and is also inhibited in HTLV-1 infected T-cell lines. Knockdown of HBZ increases Bim expression, indicating that Bim is a target of HBZ [61]. FoxO3a is an important transcription factor that controls the expression of Bim and FasL. Further investigation showed that HBZ forms a ternary complex with FoxO3a and 14-3-3. By doing so, HBZ impairs the DNA-binding ability of FoxO3a and also sequesters inactive phosphorylated FoxO3a in the nucleus, thereby repressing the transactivation of Bim and FasL by FoxO3a [61]. Interestingly, FoxO3a is also a target of Tax in anti-apoptosis and CD4 T-cell persistence [62], suggesting an important role of FoxO3a in regulating apoptosis in HTLV-1 infected cells.

Autophagy is a natural cellular digestion mechanism that removes unnecessary or damaged cellular components [63]. It can be routinely triggered by amino acid deprivation, which also inhibits the activity of the mammalian target of rapamycin (mTOR) complex 1 (mTORC1) [63]. mTORC1 is a signaling complex of the mTOR pathway, which regulates cellular metabolism and promotes cell proliferation in response to proper environmental stimuli [63]. Autophagy and the mTOR pathway are inversely coupled, and mTOR inhibition has been shown to induce autophagy [63]. Recently, HBZ was reported to activate the mTOR pathway via interacting with and inhibiting growth arrest and DNA damage-inducible protein 34 (GADD34), a stress-induced GADD family protein that inhibits the mTOR pathway [64]. More importantly, starvation-induced autophagy was suppressed by HBZ, which might be due to its activation of the mTOR pathway [64]. However, more direct evidence is needed to prove this possibility. In addition, GADD34 itself is involved in the regulation of apoptosis [65], so it would be interesting to further evaluate the possible impact of HBZ/GADD34 interaction on apoptosis.

#### HBZ disrupts genomic integrity

Genomic instability is a hallmark of cancer, and various kinds of genetic alterations have been reported in ATL [21]. A recent study found that HBZ expression induces double strand breaks (DSBs) in transfected HeLa cells, and is the first to link HBZ to genomic instability [66]. Intriguingly, HBZ-induced DSBs are dependent on several microRNAs (miR) that are HBZ-inducible, such as miR17 and miR21. miR17 and miR21 target and suppress the expression of OBFC2A, the gene that encodes hSSB2, a single-stranded DNA-binding protein that prevents genomic instability. Overexpression of OBFC2A counteracts the DNA-damaging effect of HBZ [66]. It is thus proposed that HBZ disrupts host genomic integrity through this HBZ-microRNA-OBFC2A cascade.

Telomeres are chromosomal regions composed of tandem repeats of TTAGGG and are localized at chromosomal ends [67]. It is well known that telomeres become shortened after each cell division, until cells reach a state of replication senescence known as the “Hayflick limit.” Telomeres can be replenished by telomerase, which contains an important catalytic unit called telomerase reverse transcriptase (TERT) whose activity is kept low in normal somatic cells [67]. In contrast, cancer cells often break the “Hayflick limit” by elevating TERT expression to allow sustained proliferation. HBZ has been reported to promote human TERT (hTERT) expression via enhancing its promoter activity in association with JunD [68]. The activities of two inhibitors of the hTERT promoter, TAL1 and menin, are also suppressed by HBZ [69, 70]. Elevation of hTERT levels by HBZ may allow sustained proliferation of ATL cells, which have been reported to overexpress hTERT [71, 72].

#### HBZ induces inflammation

HBZ-transgenic (Tg) mice, in which HBZ is expressed only in CD4+ T cells, frequently develop dermatitis, and some of HBZ-Tg mice develop lymphoma [73–75]. In HBZ-Tg mice, increased numbers of Foxp3+CD4+ T cells were found. This population contains regulatory T cells (Tregs), a T-cell subset known to suppress effector T cells [38]. HBZ induces T cells to become Tregs by enhancing the TGF-β/Smad pathway via forming a ternary complex with Smad3/p300 and thereby upregulating expression of Foxp3, the TGF-β-inducible master transcription factor of Tregs [38]. However, Foxp3 expression is unstable in Foxp3+ T cells of HBZ-Tg mice, and thus Foxp3+ T cells convert to Foxp3− T cells with enhanced production of IFN-γ [74]. This enhanced production of IFN-γ is associated with both inflammation and the development of lymphomas in HBZ-Tg mice; loss of IFN-γ suppresses both of these phenomena [75]. In addition, the ability to induce Treg differentiation and inflammation development is completely an intrinsic characteristic of HBZ, because even HBZ-Tg mice maintained in a germ-free environment had the same phenotype as those raised in a normal specific-pathogen-free environment [75], excluding the role of extrinsic factors such as the gut microbiota.

Another interesting finding is that HBZ-Tg mice exhibited impaired immune responses to herpes simplex virus or *Listeria monocytogenes* infection compared to WT mice [76]. Th1 cytokine production was significantly reduced in infected HBZ-Tg mice, an observation which seems attributable to HBZ-mediated NFAT and AP1 inhibition. The compromised immune response of HBZ-Tg mice to infections likely correlates with the observation that some ATL patients suffer from opportunistic infections and implies a role of HBZ in the impaired immunity of ATL patients [76].

#### HBZ protein is low immunogenic

Immunogenicity is the ability of a foreign antigen/epitope to provoke a cellular or humoral immune response in the host. It is a key issue in vaccine development that greatly affects vaccine efficiency. Among all HTLV-1 viral proteins, HBZ likely has the lowest immunogenicity, because anti-HBZ antibodies could rarely be detected in HTLV-1 infected individuals, whereas antibody responses to other viral antigens could be easily detected. Nevertheless, an HBZ-specific humoral response has been detected by a highly sensitive luciferase immunoprecipitation system (LIPS) [77, 78]. Interestingly, anti-HBZ serum from one HAM/TSP patient inhibited the proliferation of CD8 T cells from an HBZ-antibody-negative HAM/TSP patient [77]. However, whether anti-HBZ antibody is truly inhibitory to HBZ-expressing infected cells remains to be clarified by future studies.

Compared to the anti-HBZ humoral response, HBZ-specific cellular immunity has been studied more extensively. Hilburn et al. [79] detected HBZ-specific CD4 or CD8 T cells in only about half of asymptomatic HTLV-1 carriers and HAM/TSP patients tested and found that HBZ-specific CTLs are associated with low proviral load and asymptomatic carriage.

The low level of expression of HBZ protein in HTLV-1 infected cells is one likely reason for the inefficiency of the specific immune response. In accordance with this notion, another study that compared the lysis of HTLV-1 infected cells by HBZ- versus Tax-specific CTLs attributed the unsatisfactory lytic efficiency of HBZ-specific CTLs to inadequate presentation of HBZ epitopes [80]. Therefore, despite the fact that HLA I binding of HBZ provides the biggest protective/detrimental ratio as suggested by in vitro experiments and computational analysis [81], the relatively weak binding strength of HBZ epitopes to CTLs in vitro as well as the low expression of HBZ protein in vivo might greatly hinder the ability of the host to mount a successful anti-HBZ CTL response. Nevertheless, a recent study suggests that mice and macaques that have been immunized by a recombinant vaccinia virus-based HBZ vaccine can successfully generate HBZ-specific CD4 and CD8 T-cell responses [82, 83]. Importantly, anti-HBZ CTLs from immunized mice are protective when adoptively transferred to an HBZ-induced ATL mouse model [83]. Furthermore, a candidate HBZ peptide (157-176 aa) was identified for future human vaccine development [83]. Nonetheless, low immunogenicity of HBZ likely facilitates infected cells’ evasion of immunosurveillance and perhaps contributes to the HTLV-1-mediated oncogenesis.

## *HBZ* RNA is growth promoting and anti-apoptotic

HBZ is unique in that it is the only HTLV-1 gene transcribed from the antisense strand—a subtle mechanism that avoids the use of the frequently disrupted or methylated 5′ LTR as its promoter and also evades host APOBEC3G-induced nonsense mutations [18, 20]. Intriguingly, *HBZ* RNA carries regulatory functions aside from the common protein-coding function. It has been reported that *HBZ* RNA itself supports the proliferation of the IL-2-dependent T-cell line Kit225 cultured at a suboptimal concentration of IL-2. The precise mechanism is unclear but *HBZ* RNA likely achieves this by targeting E2F1 and upregulating its expression, as well as the expression of its downstream target genes, to enhance proliferation [25]. A recent study revealed that *HBZ* RNA also inhibits apoptosis in mouse CD4 T cells [84]. *HBZ* RNA increases the transcription of the anti-apoptotic gene *survivin*, a fact which likely accounts for its anti-apoptotic effects. These two key functions of *HBZ* mRNA (proliferation-enhancing and anti-apoptotic) imply that HBZ might contribute to the oncogenesis of ATL in its RNA form as well. Since mRNAs ordinarily localize to the cytoplasm for the sake of translation, whereas regulatory long noncoding RNAs (lncRNA) are found mostly in the nucleus [85, 86], the dominant nuclear localization of *HBZ* RNA reported in several studies [54, 55, 87] supports the regulatory role of *HBZ* RNA as well.

## Antisense protein of HTLV-2 (APH-2)

HTLV-2 is a close relative of HTLV-1 that has not been linked to any diseases and thus is considered to be non-pathogenic. HTLV-2 has also been reported to encode an antisense transcript termed APH-2 [88]. APH-2 has similar functions as HBZ such as inhibiting transcription from HTLV-2 5′ LTR and is dispensable for in vitro T-cell immortalization [89]. APH-2 demonstrates distinct activities as HBZ in modulating cellular pathways [90] whereas it lacks the ability to promote T-cell proliferation [91], which might contribute to the nonpathogenic nature of HTLV-2.

## Perspective

**Fig. 3:**
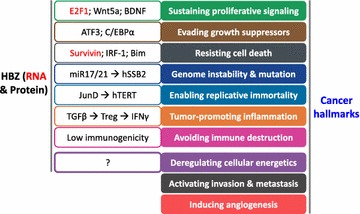
HBZ viewed from a cancer hallmarks perspective. Ten cancer hallmarks are listed and painted in *different colors* on the *right* [92]. *White boxes* on the *left* briefly outline functions of HBZ that relate to the corresponding cancer hallmark on the right. HBZ RNA-related functions are marked in *red*

It has been assumed as an oncogenesis model for HTLV-1 that Tax initiates cellular transformation at an early stage, while HBZ maintains the transformed phenotype during the late stage when Tax expression is absent or suppressed. However, since HBZ expression has always been present regardless of early or late stage, it seems inaccurate to attribute the first hit of oncogenesis solely to Tax. In fact, accumulating evidence now implies a critical role of HBZ throughout the course of HTLV-1 mediated oncogenesis. As summarized above, HBZ has been found to carry a wide variety of functions that associate with seven out of ten cancer hallmarks (Fig. 3) [92]. If we exclude hallmarks like angiogenesis or invasion that are generally characteristics of solid cancers, then deregulation of cellular energetics is the only hallmark yet to be associated with HBZ. Hence, despite the fact that HBZ has not been reported to associate directly with cellular transformation like Tax does, HBZ does seem to harbor the required potential to cause cancer. Tax and HBZ frequently shows opposite effects on signaling pathways, suggesting that HBZ plays different roles in oncogenesis from Tax. Furthermore, given the fact that the HBZ gene is the only HTLV-1 gene present in all infected individuals, HBZ-targeting strategies are expected to serve as promising therapeutics for ATL in the future.
